# Dropped Gallstone Mimicking Retroperitoneal Tumor 5 Years After Laparoscopic Cholecystectomy Posing a Diagnostic Challenge

**DOI:** 10.7759/cureus.31284

**Published:** 2022-11-09

**Authors:** Prabasha Weeraddana, Niwanthi Weerasooriya, Teena Thomas, Joseph Fiorito

**Affiliations:** 1 Internal Medicine, Danbury Hospital, Danbury, USA; 2 Gastroenterology, Danbury Hospital, Danbury, USA

**Keywords:** spilled, complication, ct abdomen, retroperitoneal abscess, laparoscopic cholecystectomy, dropped gallstones

## Abstract

Laparoscopic cholecystectomy is the standard treatment for cholecystitis. The major complications associated with laparoscopic cholecystectomy include bleeding, abscess formation, biliary injury with bile leakage, and bowel injury. Gallbladder perforation and subsequent stone spillage are not uncommon in laparoscopic cholecystectomy. The majority of these spilled stones remain clinically silent. But sometimes they can cause abscesses and make diagnosis challenging especially when it occurs years after the procedure and when the abscess form in uncommon sites. A 66-year-old female with a history of laparoscopic cholecystectomy presented with aggravating abdominal pain in the right upper quadrant (RUQ). The CT abdomen revealed a mass in the retroperitoneum behind the hepatic flexure. Upon further examination, follow-up CT scans, and biopsy repeatedly failed to exclude malignancy, so it was suggested that the patient undergo surgical removal of the mass. The pathological analysis of the excised mass revealed that it was a dropped gallstone from the procedure that triggered an inflammatory reaction. Dropped gallstones should be considered as a differential diagnosis in a patient with a history of laparoscopic cholecystectomy presenting with an abdominal or retroperitoneal abscess, as a failure of early recognition puts the patient at risk of undergoing unnecessary and invasive procedures.

## Introduction

Cholelithiasis is estimated to affect 15% of the adult population [1]. Symptomatic gallbladder disease is currently treated with laparoscopic cholecystectomy (LC), which is the accepted standard of treatment [2]. LC has a higher incidence of gallbladder perforation and subsequent stone spillage than open cholecystectomy, with rates ranging from 8 to 30% of all procedures [3]. However, gallstones rarely drop into the retroperitoneum. The body treats gallstones as foreign particles and causes an inflammatory response, leading to the symptoms [4]. We present a case where lost gallstones were found in the retroperitoneum and were surgically excised when an inflammatory reaction resulted in the symptoms of aggravating abdominal pain.

## Case presentation

A 66-year-old patient with a significant past medical history of choledocholithiasis for which she underwent endoscopic retrograde cholangiopancreatography (ERCP) with stone removal and LC 5 years back presents to our hospital with constant dull right upper quadrant (RUQ) abdominal pain. She had several ED visits for episodes of intermittent severe RUQ pain during the last 3-4 months. She denied fevers, nausea, vomiting, or changes in bowel function. On physical exam, the patient’s vital signs were within normal range. Abdominal examination revealed a non-distended abdomen with tenderness in the RUQ and right flank, no palpable organomegaly, no masses, and normal bowel sounds. She had normal WBC and Hb levels with a marginally low platelet count of 139 platelets per µl. Her bilirubin, liver function tests, and alkaline phosphatase levels were within normal limits. Urinalysis showed no hematuria or other abnormalities.

**Figure 1:**
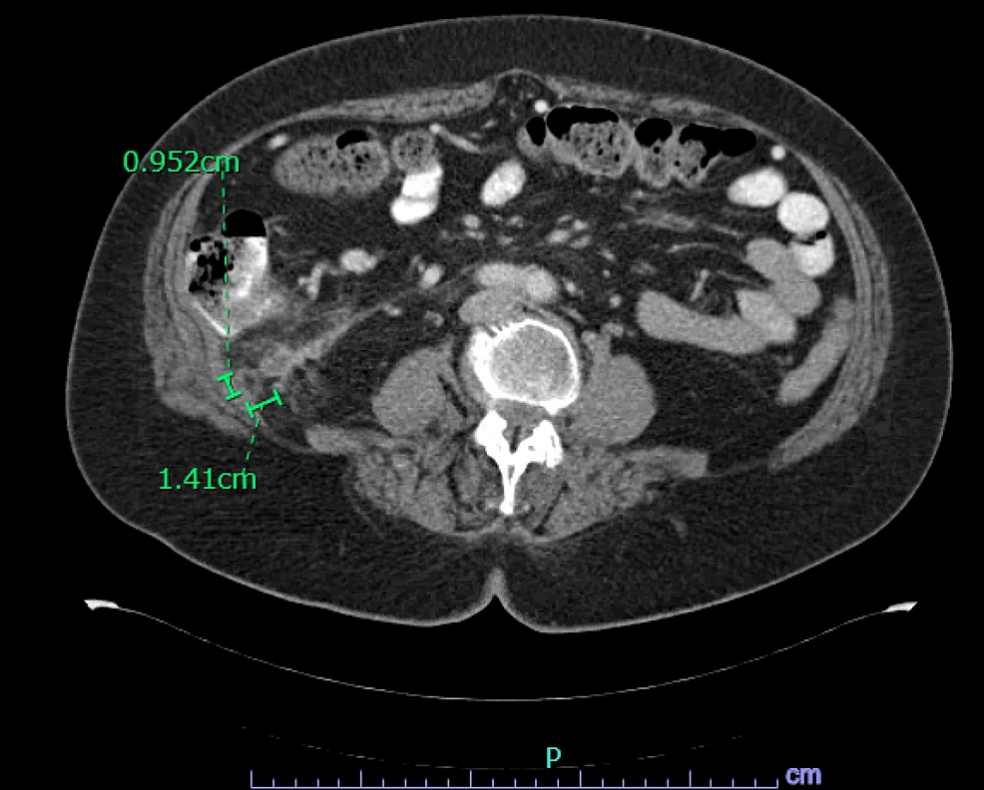
CT abdomen and pelvis with contrast showing posterior to the hepatic flexure along the inferior aspect of the right hepatic lobe. There is a focal area of fat stranding with an associated fluid collection measuring 1.4 x 1.0 x 1.2 cm. There is no significant colonic wall thickening.

**Figure 2:**
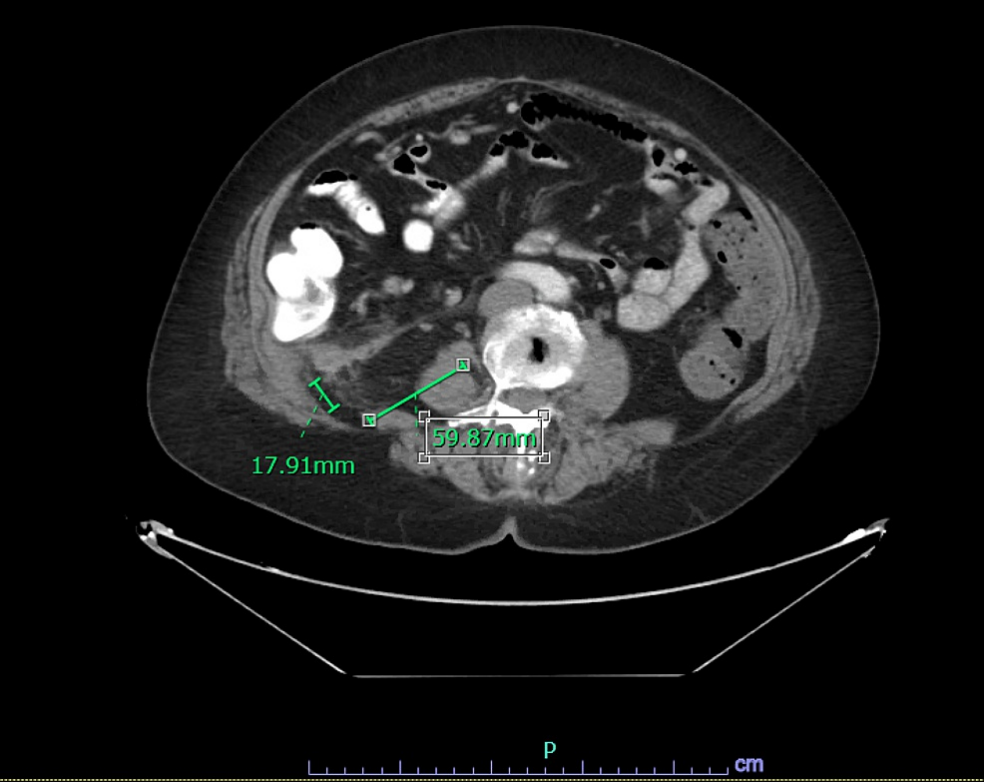
CT chest abdomen and pelvis show abnormal fat stranding adjacent to the hepatic flexure of the colon with retroperitoneal soft tissue mass measuring 5.9 x 1.7 cm in greatest transverse dimensions adjacent to infiltration.

She was previously evaluated by her primary care physician for abdominal pain and obtained a CT abdomen as an outpatient. The CT abdomen and pelvis with contrast revealed a focal area of fat stranding adjacent to the hepatic flexure and along the inferior aspect of the right hepatic lobe with an associated focal fluid collection measuring 1.4 x 1.0 x 1.2 cm (Figure 1). Differential diagnoses included right-sided diverticulitis, omental infarct, or epiploic appendicitis. She then had a follow-up CT abdomen, which confirmed the previous test; in addition, a soft tissue component measuring 5.9 x 1.7 cm (Figure 2) in the greatest transverse dimensions adjacent infiltration was noted. The soft mass appeared separate and distinct from the adjacent colon and was located along the lower margin of Gerota’s fascia on the right, appearing contiguous with the fascia.

**Figure 3:**
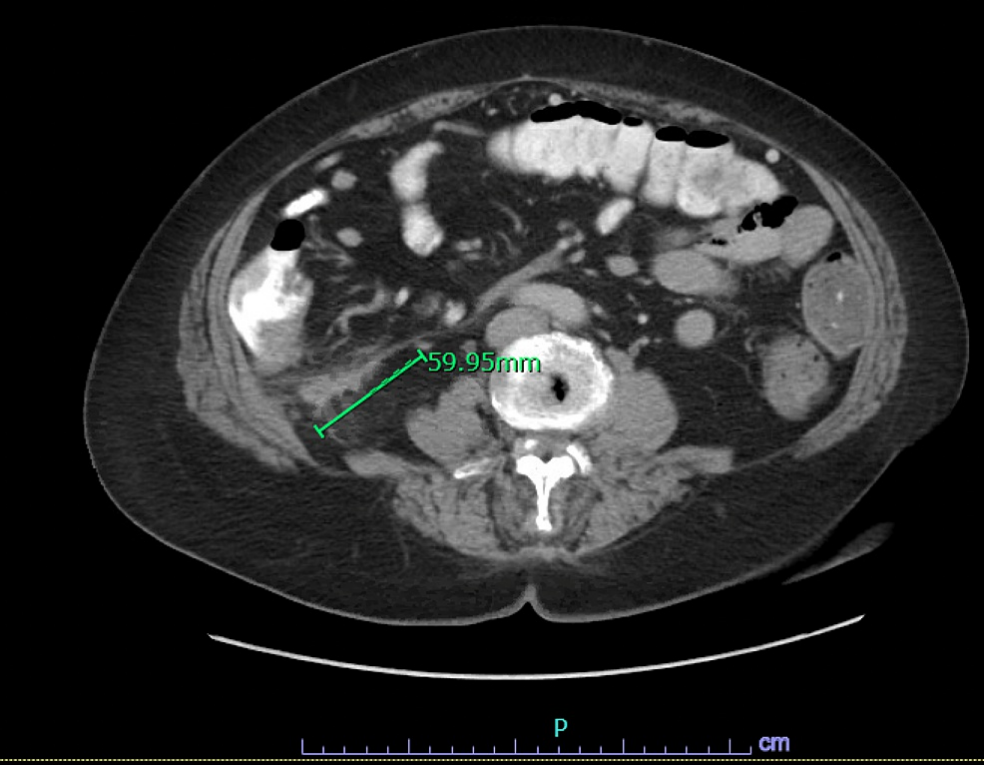
Repeat CT chest abdomen and pelvis show grossly stable 5.9 x 1.7 cm nodular abnormal soft tissue density within the right upper quadrant retroperitoneum posterior to the hepatic flexure.

**Figure 4:**
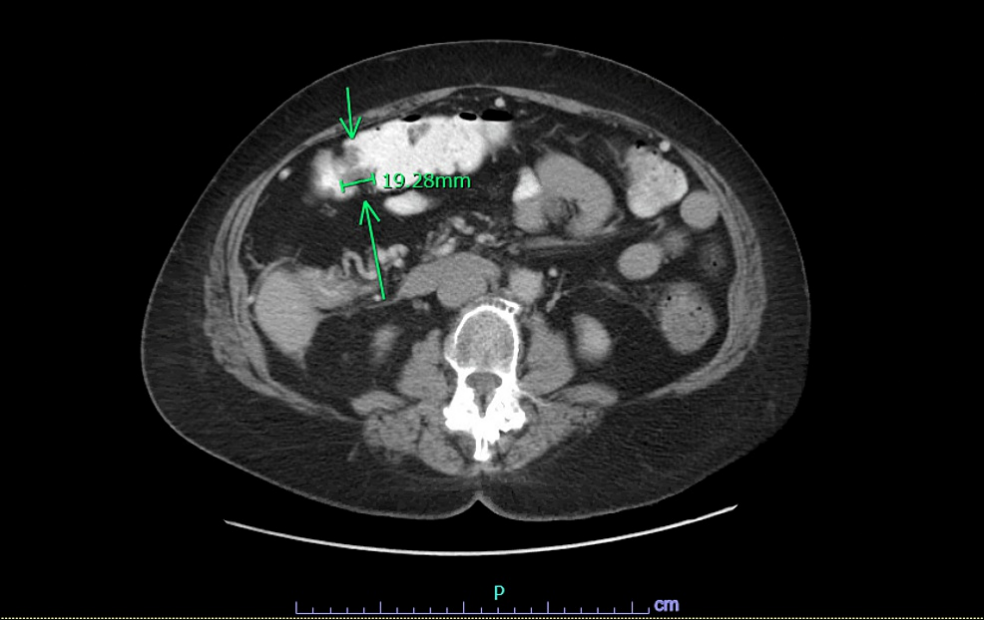
Repeat CT chest abdomen and pelvis shows a 1.9 cm concentric area of wall thickening involving the proximal transverse colon.

**Figure 5:**
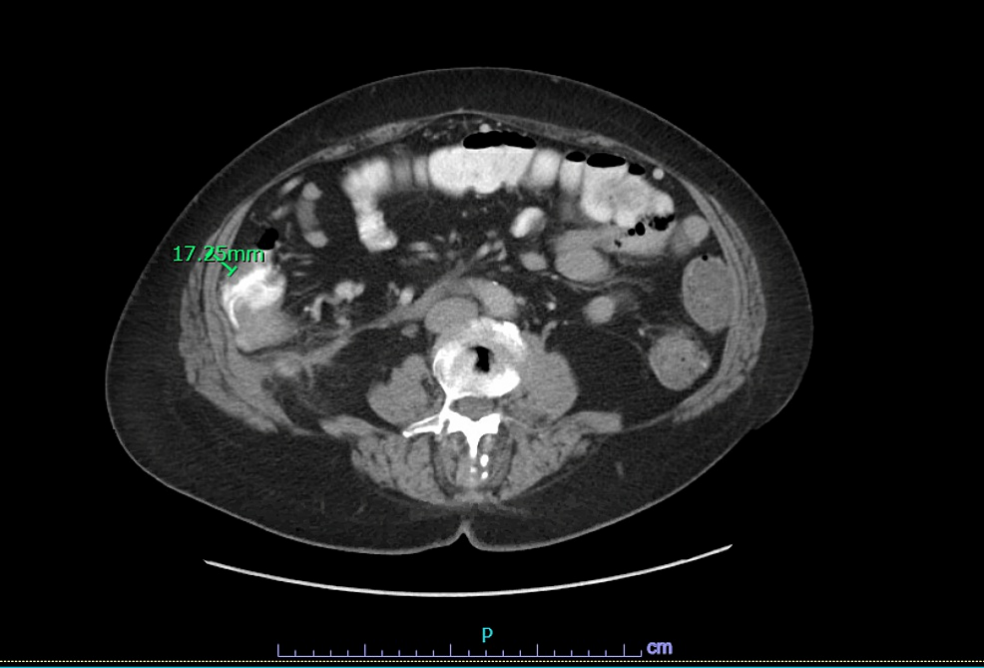
Additional areas of wall thickening involving the hepatic flexure.

She was started on antibiotics (cefdinir) for a week. She underwent colonoscopy and upper endoscopy, which showed chronic gastritis and a few hyperplastic polyps. The findings were negative for Barrett’s esophagus and colitis. She had a follow-up CT abdomen in 4 weeks to see if there was resolution or progression, and it showed grossly stable abnormal nodular soft tissue density, measuring 5.9 x 1.7 cm, within the RUQ, posterior to the hepatic flexure in the retroperitoneum (Figure 3). The CT also revealed a possibly slightly lobular liver contour and intra-abdominal varices along with splenomegaly. After further evaluation, she was scheduled for an IR-guided biopsy and sampling. Meanwhile, she had worsening abdominal pain and was admitted to the ED where she had another CT abdomen which showed a 1.9 cm concentric area of wall thickening involving the proximal transverse colon (Figure 4). Additional areas of wall thickening involved the hepatic flexure (Figure 5). Although the findings were suspected to be due to focal colitis, the possibility of underlying malignancy could not be excluded.

**Figure 6:**
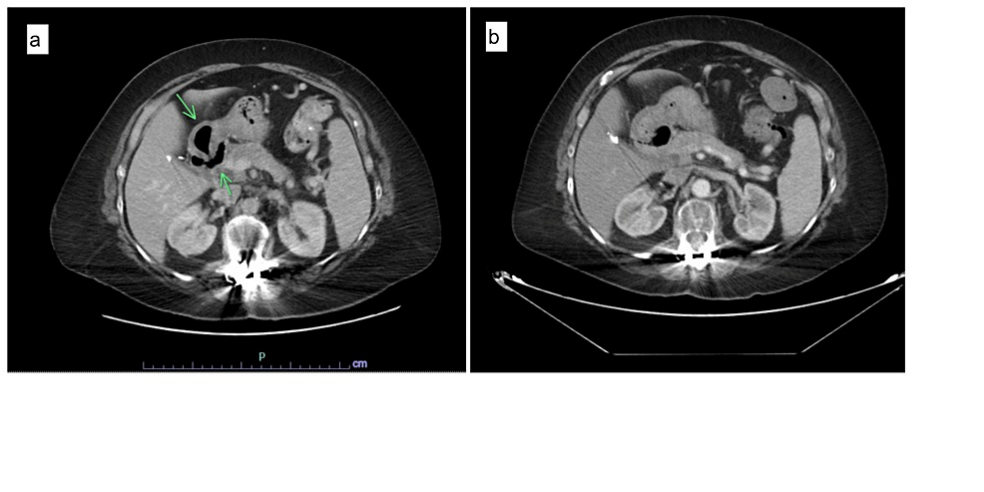
CT abdomen and pelvis demonstrate apple core lesion involving hepatic flexure of the colon (a). Normal hepatic flexure of the colon in previous CT abdomen for comparison (b).

The nodular abnormal soft tissue density within the RUQ retroperitoneum posterior to the hepatic flexure was unchanged. After a couple of days, she underwent a CT-guided biopsy, which showed chronic inflammatory changes in the right retro colic space, which was probably related to a dropped gallstone. Moreover, it showed fibrous tissue infiltrated with mixed acute and chronic inflammatory cells. The result was negative for malignancy. Then the patient was referred to surgery. She had ongoing pain, ranging from 6 to 7 out of 10. Despite a course of antibiotics, the symptoms failed to improve. One of the CT scans did show what looks like an apple core lesion in her proximal transverse colon (Figure 6), although she underwent a colonoscopy and had no obvious findings of malignancy in this area.

She was subsequently evaluated by oncology surgery for persistent abdominal pain with worsening RUQ inflammatory changes along with a persistent retroperitoneal mass; hepatic flexure of the colon now appears to have an apple core lesion on the CT scan. Then she underwent CT enterography to rule out Crohn’s disease. This showed the persistence of the retroperitoneal soft tissue lesion. Areas of bowel wall thickening previously seen in the transverse colon and ascending colon were no longer present. She underwent retroperitoneal exploration with tumor resection. Intraoperatively, the patient was found to have an abscess posterior to the duodenum. The duodenum was densely adherent to the retroperitoneum. The fluid in the abscess cavity was swabbed and sent off for culture and gram stain. There was a large mass in the retroperitoneum at the inferior border of the right kidney. The mass was circumferentially dissected. During this dissection, we encountered a stone within the cavity. The stone appeared to be a retained gallstone. The stone was also removed and sent separately for examination. The mass was then excised and passed off to the frozen section.

**Figure 7:**
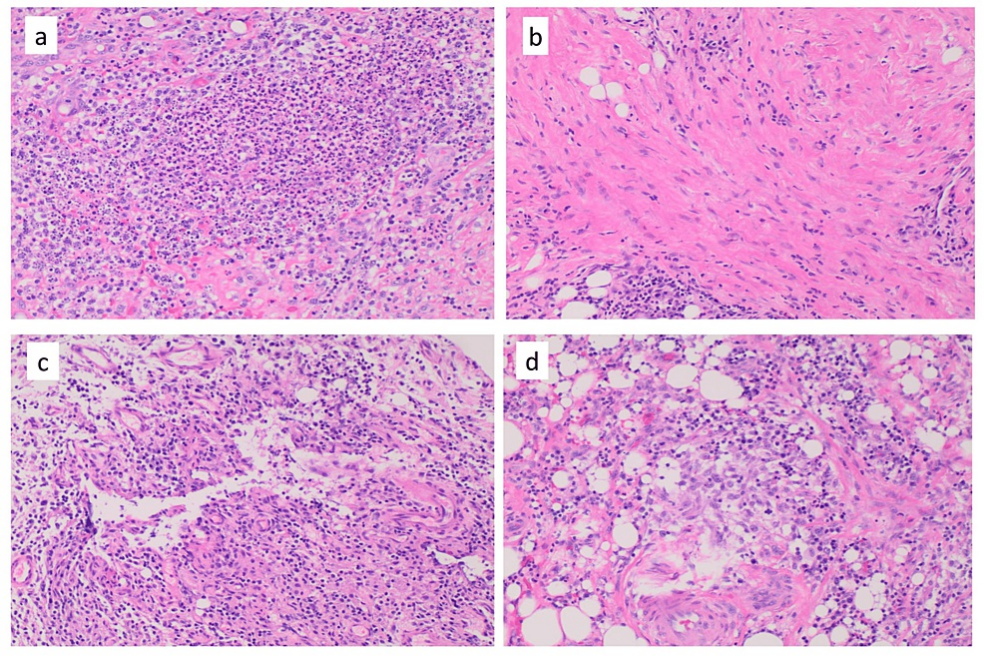
a) Acute and chronic inflammation, H&E, 10x; b) fibrosis, H&E, 10x; c) granulation tissue, H&E, 10x; d) granuloma, H&E, 10x.

**Figure 8:**
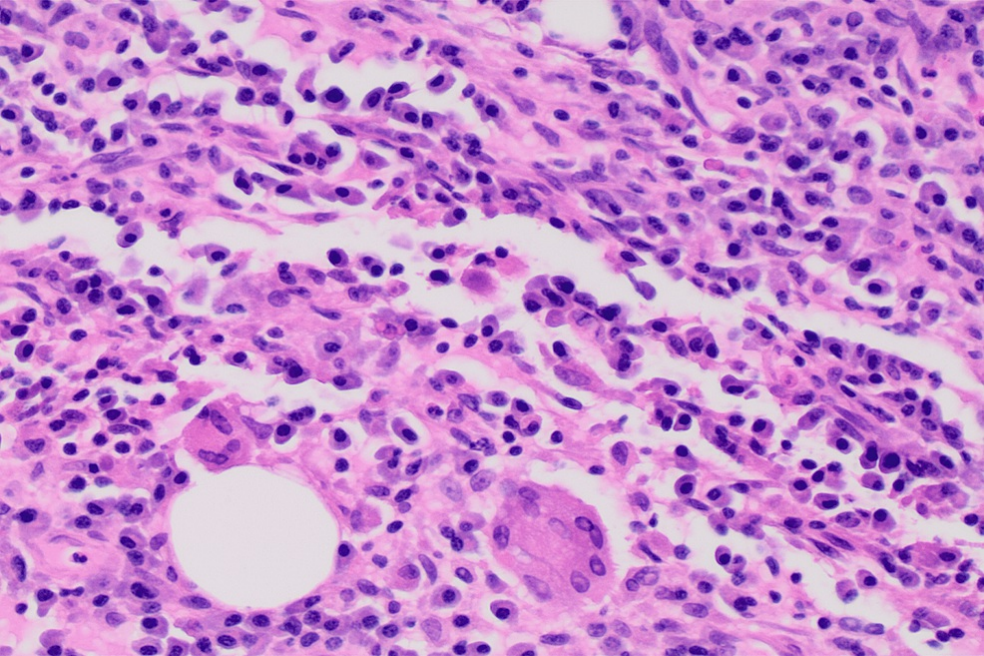
Foreign body giant cell reaction, H&E, 20x.

The pathological examination of the excised right-sided retroperitoneal mass revealed fibrosis and granulation tissue suggestive of acute, chronic, and non-necrotizing granulomatous inflammation (Figure 7) and foreign body giant cell reaction (Figure 8). The report confirmed non-malignancy and established the gross diagnosis of dropped gallstones in the retroperitoneum. The retroperitoneum was then irrigated and a drain was placed. Its output and consistency were regularly monitored until it was low volume and serous before removal. After surgery, her Hb dropped to 7.3 mg/dL, for which she received two units of packed red blood cells (PRBC). During hospitalization, she was given IV ceftriaxone and metronidazole. There was no growth in intraoperative abscess cultures. She was discharged with a prescription for Augmentin for 1 week. The patient remained well without major complications after 1 month of follow-up.

## Discussion

Cholelithiasis is a common presentation in hospitals worldwide and affects 10-15% of the world’s population [1]. Gallstones are removed surgically by either open or LC. Cholecystectomy has become the most common elective abdominal surgery. LC is preferred over the open approach due to early healing and better outcome, but it has its own set of complications. During this procedure, perforation of the gallbladder can occur, resulting in the spillage of gallstones into the abdominal cavity. Spilled gallstones can result in intra-abdominal infections and sometimes form retroperitoneal abscesses. The incidence of these complications ranges from 8 to 30% of all cases [5].

The gallstones are mostly lost in the peritoneal cavity, but pneumoperitoneum and irrigation of the peritoneal cavity disperse gallstones to multiple sites, i.e., subhepatic space, right flank, right subphrenic, and even retroperitoneal spaces. These spilled gallstones are mostly asymptomatic but sometimes lead to complications. The clinical presentation of complications can vary according to the site and size of the dropped gallstone. They may present with abdominal complaints like pain, mass, fever, obstruction, and fistulas [6]. These lost gallstones might be reabsorbed but mostly remain intact and can lead to the formation of abscesses. Some other rare complications of spilled gallstones include obstructive cholangitis, secondary acute appendicitis, intestinal obstruction, and volvulus [6]. The most common site of abscesses masses containing the spilled gallstone is subhepatic space [7]. However, rarely it can cause abscess formation in retroperitoneal space as in our case.

The risk of gallbladder perforation during cholecystectomy was studied by De Simone et al. They figured out three risk factors, i.e., previous laparotomy, bladder wall thickness >7 mm, and non-decompressed hydropic gallbladder. The risk of perforation is 25% with all these risk factors present [8]. The body treats spilled gallstones as foreign objects, triggering an inflammatory reaction that may lead to local fibrosis around the stone. The body occasionally reacts with partial reabsorption of the stone. As in our case, however, spilled gallstones can also erode into the nearby peritoneum and cause an extraperitoneal inflammatory response that results in the development of an abscess [4].

Woodfield et al. conducted a review and the complication rate due to peritoneal gallstone was found to be 0-5 per 1000 LCs, with a total of 27 patients presenting with complications out of 18,280 LCs performed. 60 and 30% of these complications were intraperitoneal abscess formation and fistula formation. Other complications reported were empyema, intestinal obstruction, diaphragmatic irritation, erosion of stone through the flank, and peritoneal calculi [9].

Gallstones inside the abscess are often seen on CT and MRI scans, which is crucial for making a diagnosis [10]. While pigment stones with high calcium content are readily recognized with CT, pure cholesterol stones and those with minimal calcium concentration might go undetected. In addition, the use of contrast during CT scans will enhance the surrounding inflammation. It might conceal the stone, resulting in misdiagnosis as a tumor or other pathologies, such as retroperitoneal sarcoma, peritoneal metastases, or lymphadenopathy. The diagnosis was challenging in this case, because the gallstone abscess simulated a retroperitoneal mass in radiologic findings, resulting in delayed diagnosis [11].

LC-related abscess development has been observed to have onset within an average of 4 to 10 years. Although the diagnosis of spilled gallstones is favored by the presence of calcification, other mucin-producing tumors, such as those from the ovary and colon, may include calcium [12]. When patients have a history of LC, the presence of highly reflecting echoes with posterior shadowing in the abscess cavity may be pathognomonic for gallstone abscess.

In our case, a 66-year-old female with a surgical history of LC presented with a complaint of aggravating abdominal pain in RUQ. A CT abdomen revealed a soft mass in the retroperitoneum posterior to the hepatic flexure. Despite a course of antibiotics, the symptoms did not get any better, and follow-up CT scans showed an unchanged mass. It was finally suspected to be a dropped gallstone from the procedure she underwent five years ago. Finally, the mass was excised surgically and the pathological examination confirmed the diagnosis that it was a spilled gallstone, triggering an inflammatory reaction.

This case makes an addition to the literature on dropped gallstones by concluding that CT scans are not quite useful in making a diagnosis of dropped gallstones in the retroperitoneum. Despite various follow-up CT scans, the case remained undiagnosed until the pathological examination of the excised mass revealed the spilled gallstone. In addition, even malignancy could not be excluded from either the laboratory findings or the scans, which resulted in extensive investigation and testing, thereby further contributing to the morbidity of the patient.

## Conclusions

Complications related to spilled gallstones are more commonly seen in LCs compared to open cholecystectomies. In some patients, these complications from dropped calculi may not appear for several years after the initial surgery. Dropped gallstones might not be taken into consideration in the differential diagnosis, because of the long duration between cholecystectomy and clinical manifestation, which could result in an incorrect diagnosis, a series of futile invasive testing, resulting in ineffective management, and needless medical expenditure. Although imaging techniques such as ultrasound and CT scans are recommended for the evaluation of suspected spilled gallstones, these techniques do have their limitations. Efforts should be made to retrieve spilled stones during LC to avoid complications in the future. Investigating intraperitoneal or retroperitoneal abscess development in someone with a surgical history of cholecystectomy necessitates having a high index of suspicion for spilled gallstones for early diagnosis and treatment.
